# Antismoking Mass Media Campaigns and Support for Smoke-Free Environments, Mobile County, Alabama, 2011–2012

**DOI:** 10.5888/pcd11.140106

**Published:** 2014-09-04

**Authors:** Gabriel H. Fosson, Debra M. McCallum, Michael B. Conaway

**Affiliations:** Author Affiliations: Debra M. McCallum, Michael B. Conaway, The University of Alabama, Tuscaloosa, Alabama.

## Abstract

**Introduction:**

In 2011, the Mobile County Health Department began a 12-month antismoking educational media campaign to educate citizens on the dangers of secondhand smoke. The campaign overlapped with the Centers for Disease Control and Prevention’s 3-month national antismoking Tips from Former Smokers media campaign. We aimed to evaluate the effect of these campaigns on support for smoke-free environments and knowledge of the dangers of secondhand smoke.

**Methods:**

Cross-sectional precampaign and postcampaign telephone surveys collected data from a random sample of Mobile County adults in the summers of 2011 and 2012. Outcome measures included changes in support for smoke-free environments and knowledge of the dangers of secondhand smoke. The participation rate among the households that were successfully reached was 45% in 2011 and 44% in 2012.

**Results:**

On the postcampaign survey, 80.9% of respondents reported seeing a television advertisement, 29.9% reported hearing a radio advertisement, and 49.0% reported seeing a billboard. Overall, support for smoke-free bars increased significantly after the intervention (38.1% to 43.8%; *P* = .01) but not for workplaces or restaurants. Self-reported exposure to the media campaign was associated with higher levels of support for smoke-free workplaces, restaurants, and bars.

**Conclusion:**

Educational mass media campaigns have the potential to increase support for smoke-free protections and may increase knowledge about the dangers of secondhand smoke among certain populations.

## Introduction

Educational mass media campaigns against smoking can be an effective means of changing attitudes (1–3) and behaviors (4–8) related to tobacco use. Media campaigns have the potential to change social perceptions of smoking (1,2) and knowledge about the dangers of secondhand smoke (SHS) (2) and thus may increase support for smoke-free environments. However, research on the effect of educational media campaigns on desire for smoke-free protections is limited. Two such studies have been conducted within the past 5 years in Mexico City, Mexico, and in São Paulo, Brazil, evaluating the effects of media campaigns on support for recently protected smoke-free environments. Results from both studies concluded that exposure to the media messages was positively associated with increased support for the new smoke-free protections (9,10). We investigated the effect of an educational media campaign on support for smoke-free environments before the implementation of smoke-free protections.

In 2010, Mobile County, Alabama, home to 413,000 residents, had a higher prevalence of smoking (25.0%) than the national average (17.3%) (11,12), and only 2 of its 12 municipalities had adopted legislation restricting smoking in indoor public places. Without a state law restricting smoking in indoor public places, municipalities in Alabama are required to enact local legislation to protect residents from SHS. Mobile County was selected in 2010 by the Centers for Disease Control and Prevention (CDC) to participate in the Communities Putting Prevention to Work (CPPW) initiative. The purpose of the national CPPW program was to reduce chronic disease associated with tobacco use or obesity through sustainable, high-impact change in systems and environments (13). The Mobile CPPW program used an antitobacco educational media campaign as one of its evidence-based interventions to help educate residents about the dangers of tobacco use and SHS exposure.

The objective of our evaluation was to assess the reach and potential effects of the Mobile County Just Breathe Smoke Free (Just Breathe) educational media campaign and CDC’s Tips from a Former Smoker (Tips) national antismoking media campaign on residents’ attitudes toward smoke-free environments and knowledge about the dangers of SHS.

## Methods

### Media campaign

The Mobile County Health Department (MCHD) contracted with Lewis Communications to manage and assist in developing the Just Breathe educational media campaign. The campaign included television, radio, print, billboard, and online advertisements. MCHD produced an upbeat, animated television advertisement based on focus group research for this campaign and ran 3 additional television advertisements borrowed from previous campaigns that focused on the dangers of SHS. Additionally, MCHD ran 2 radio, 8 print, and 3 billboard advertisements.

From September 26, 2011, to September 30, 2012, the campaign placed the following paid advertisements: 1,700 television spots, 2,100 cable spots, 8,500 radio spots, and 200 print insertions in local newspapers and magazines. Billboard advertisements were placed between September 26, 2011, and August 31, 2012, with a daily effective circulation (the average number of adults 18 or older passing and potentially exposed to an advertisement each day) of 488,000. Paid online advertising was placed between September 26, 2011, and December 12, 2011, as follows: 14.2 million total impressions (the number of times an advertisement is displayed online) on Facebook resulting in 1,900 clicks and 6.3 million impressions on AL.com resulting in 6,800 clicks. The campaign also received earned media exposure, including editorials, news stories, and public service announcements, which added unpaid advertising in each media outlet.

CDC’s Tips campaign overlapped with the Just Breathe campaign. The Tips campaign lasted for 12 weeks, starting on March 19, 2012, and included television, radio, print, billboard, and website advertisements. In contrast to the Just Breathe campaign, the Tips campaign used hard-hitting, emotional advertisements, which depicted suffering caused by smoking. An evaluation of the Tips campaign found that it was effective at increasing population-level quit attempts (14).

### Survey methods

A precampaign–postcampaign cross-sectional telephone survey was conducted by The University of Alabama to assess the effects of the educational media campaigns. Researchers at the university developed and pilot-tested survey questions to evaluate exposure to the educational media campaign and support for smoke-free policies. Questions regarding tobacco use, knowledge of the dangers of SHS, and awareness of the quitline were taken from previously validated surveys (15), including CDC’s Behavioral Risk Factor Surveillance System and Adult Tobacco Survey and previous tobacco studies conducted by the university. The baseline survey was conducted between August 8 and October 1, 2011, ending 6 days after paid advertising of the Just Breathe campaign began. The postcampaign survey occurred during the last several months of the educational media campaign from June 9 through September 5, 2012. This study was approved by the institutional review board at The University of Alabama and by the Alabama Department of Public Health.

Data collection for the study was conducted by the Capstone Poll at The University of Alabama. Random-digit dialing was used to select households in Mobile County, and in each household a respondent was randomly selected for participation. Mobile County residents aged 19 years or older were eligible to participate in the study. By using the American Association for Public Opinion Research’s response rate 1 formula (16), the participation rate in 2011 was 21.9%, based on 845 completions and 3,861 households, and in 2012 was 22.6%, based on 766 completions and 3,396 households. Approximately 52% (2011) and 49% (2012) of the telephone numbers provided were never reached after multiple attempts. Among the households that were reached, the cooperation rate was 45% in 2011 and 44% in 2012.

### Measures

Exposure to antitobacco media messages was assessed in the precampaign and postcampaign surveys using 4 questions, which asked whether participants had seen advertisements about the dangers of smoking in different media outlets: television, radio, billboard, and newspaper or other print media. In the postcampaign survey, an open-ended follow-up question after the television and radio exposure questions helped identify recalled advertisements associated with the Just Breathe or Tips campaigns. Open-ended information was not solicited about the print advertisements. Interviewers were trained to recognize 11 television advertisements (4 Just Breathe, 7 Tips) and 9 radio advertisements (2 Just Breathe, 7 Tips), coding the open-ended responses using computer-assisted telephone interviewing software during the interview.

Respondents were asked 4 questions in both precampaign and postcampaign surveys to gauge attitudes toward smoke-free policies in workplaces, restaurants, and bars and a comprehensive policy for all public indoor areas. Knowledge about the dangers of SHS was assessed using 6 questions that came from CDC’s Adult Tobacco Survey. Participants were asked to rate the overall harmfulness of SHS to health and indicate whether breathing SHS causes lung cancer in adults, heart disease in adults, colon cancer in adults, respiratory problems in children, and sudden infant death syndrome.

### Statistical methods

Poststratification weights were calculated by raking along dimensions of age, sex, race, and education, using census-based proportions to correct for the underrepresentation or overrepresentation of demographic subgroups in the data. Descriptive statistics and binary logistic regression analyses were conducted using SPSS software version 20 (IBM Inc). Yates χ^2^ tests of significance, corrected for continuity and for multiple comparisons (using Holm-Bonferroni) (17) were used to compare categorical survey responses between the precampaign and postcampaign surveys and to compare the demographic characteristics. Multivariate logistic regression was used to evaluate the effect of media exposure on support for smoke-free environments and knowledge about the dangers of SHS while controlling for smoking status, sex, race, and education level. For the regression analysis, media exposure was defined by whether respondents were able to describe 1 of the Just Breathe or Tips campaign advertisements unaided. Dichotomous categorical variables were established for questions about support for smoke-free environments and knowledge, with responses that expressed uncertainty such as “it depends” or “don’t know” being combined with the “no” responses.

## Results

There were no significant demographic differences between the unweighted precampaign and postcampaign samples (Table 1).

**Table 1:** Characteristics of Residents Surveyed Before or After (Cross-Sectional Design) an Educational Media Campaign^a^ About the Dangers of Secondhand Smoke Exposure, Mobile County, Alabama, 2011 and 2012^b^

Characteristic	Precampaign Unweighted No. (%) (n = 845)	Postcampaign Unweighted, No. (%) (n = 766)	Precampaign to Postcampaign χ^2^	*P* Value	Weighted %^c^
**Age, y**					
18–24	21 (3)	23 (3)	6.60	.36	13
25–34	54 (6)	50 (7)			17
35–44	74 (9)	84 (11)			17
45–54	149 (18)	139 (18)			20
55–64	233 (28)	171 (23)			16
65–74	178 (21)	163 (21)			10
≥75	131 (16)	129 (17)			8
**Sex**					
Male	265 (31)	253 (33)	0.44	.51	47
Female	580 (69)	513 (67)			53
**Race**					
White	552 (66)	512 (67)	3.27	.19	64
Black	249 (30)	228 (30)			32
Other	40 (5)	22 (3)			4
**Education**					
Less than high school graduate	73 (9)	69 (9)	1.01	.80	18
High school graduate	281 (33)	247 (32)			33
Some college	246 (29)	240 (31)			31
College graduate or higher	245 (29)	210 (27)			18

a The precampaign survey was conducted August–October 2011, and the postcampaign survey was conducted June–September 2012.

b Numbers may not equal total n because of missing data. Percentages may not total 100 because of rounding.

c Precampaign and postcampaign groups combined.

### Exposure to the educational media campaign

On the basis of the weighted 2012 postcampaign survey results, 80.9% of survey participants reported seeing an advertisement on television, and 29.9% of participants reported hearing an advertisement on the radio, about the dangers of smoking during the 30 days before the survey. Almost half (49.0%) of participants reported seeing a billboard, and 23.0% of participants saw advertisements in print media about the dangers of smoking during the 30 days before the survey (Table 2). Significant differences in self-reported media exposure between black and white residents were found for television advertisements (black, 86.5%; white, 77.7%; *P* = .03), but not for radio, print, or billboard advertisements.

**Table 2:** Exposure to Advertisements About the Dangers of Smoking, Support for Smoke-Free Policies, and Knowledge of the Dangers of Secondhand Smoke (SHS) Before and After an Educational Media Campaign, Mobile County, Alabama, 2011 and 2012

Survey Question	Black, %			White, %			Total, %^a^		
	Precampaign (n = 249)	Postcampaign (n = 228)	*P* Value^b^	Precampaign (n = 552)	Postcampaign (n = 512)	*P* Value^b^	Precampaign (n = 845)	Postcampaign (n = 766)	*P* Value^b^
**Media exposure — In the past 30 days. . .**									
. . . saw a TV advertisement	62.6	86.5	*<*.001	52.3	77.7	<.001	55.8	80.9	*<*.001
. . . heard a radio advertisement	25.7	34.0	.08	23.6	29.1	.06	24.8	29.9	.09
. . . saw a billboard	40.7	47.8	.11	35.7	49.0	*<*.001	36.8	49.0	*<*.001
. . . saw a newspaper or print advertisement	27.5	19.2	.03	22.1	23.2	.76	23.8	23.0	.74
**Support for smoke-free policies**									
Workplaces	80.3	81.6	.92	78.1	84.0	.16	79.3	83.0	.37
Restaurants	83.2	89.0	.04	82.6	83.2	.82	83.3	83.6	.92
Bars	40.9	49.2	.18	34.2	41.4	.02	38.1	43.8	.01
Comprehensive protections for all public indoor areas	72.1	78.3	.23	70.8	70.5	.69	72.0	71.9	.56
**Believe SHS is very harmful^c^**	71.1	86.5	<.001	61.8	61.7	>.99	65.8	71.0	.03
**Believe SHS causes . . .^c^**									
. . . lung cancer in adults	84.7	93.9	<.001	80.7	78.5	.63	82.2	84.1	.91
. . . heart disease in adults	75.8	84.8	.01	69.1	75.0	.40	71.2	78.5	<.001
. . . colon cancer in adults	36.8	41.0	.07	24.3	18.9	.09	27.9	27.5	.31
. . . respiratory problems in children	91.4	98.4	<.001	90.2	92.0	.68	90.8	94.3	.02
. . . sudden infant death syndrome	44.6	55.1	.03	31.3	29.3	.67	36.3	38.9	.30

a Includes all respondents, regardless of racial group.

b χ^2^ test of the difference between precampaign and postcampaign.

c A Holm-Bonferroni correction (17) was used for the knowledge questions to control for inflated error rates caused by multiple comparisons. On the basis of this correction, comparisons ≤.01 are significant.

When asked to describe the television, radio, and billboard advertisements they remembered, 14.1% of survey respondents described a Just Breathe advertisement (and not a Tips advertisement), 29.6% described a Tips advertisement (and not a Just Breathe advertisement), and 7.7% described advertisements from both campaigns. Overall, 51.4% of respondents were able to describe at least 1 television advertisement from either campaign.

For the radio advertisements, 9.6% of participants described a Just Breathe radio advertisement and 2.7% of participants described a Tips radio advertisement. Open-ended descriptions of the billboards suggest that approximately 13% of all respondents saw a Just Breathe billboard.

Because the Just Breathe paid advertising began 6 days before completion of the precampaign survey, we compared responses of the individuals completing the survey before and after September 25, 2011, for questions regarding media exposure and found no significant differences.

### Changes in support and knowledge

Support for smoke-free bars increased significantly from precampaign to postcampaign (precampaign, 38.1%; postcampaign, 43.8%; *P* = .01). However, there were not significant changes in support for smoke-free workplaces, restaurants, or comprehensive protections for all public indoor areas. Stratified by race, there was a significant increase in support for smoke-free restaurants among black respondents (precampaign 83.2%; postcampaign 89.0%; *P* = .04) and for smoke-free bars among white respondents (precampaign, 34.2%; postcampaign, 41.4%; *P* = .02) (Table 2).

Knowledge of the dangers of SHS increased significantly for only 1 out of 6 measures. Belief that SHS causes heart disease in adults increased from 71.2% to 78.5% (*P* < .001). However, a stratified analysis showed significant differences in changes in knowledge based on racial group and suggests that the increases in knowledge occurred primarily among black respondents with small, nonsignificant increases among white respondents. For black respondents, we found significant increases in 4 of 6 measures: the belief that SHS is very harmful and that SHS causes lung cancer in adults, heart disease in adults, and respiratory problems in children (Table 2). There were no significant increases in knowledge among white respondents.

### Campaign exposure, support for smoke-free environments, and knowledge

The multivariate logistic regression analyses showed that exposure to the educational media campaigns was associated with greater support for smoke-free environments (Table 3). Residents who reported exposure to 1 or both of the campaigns were significantly more likely than unexposed residents to support smoke-free workplaces, restaurants, and bars (*P* = .01 for each location). Among the other predictors included in our analysis, we found that nonsmokers were significantly more likely than smokers to support all of the smoke-free environments (*P* < .001), and that respondents with at least some college education were more likely than those with less than a high school education to support smoke-free workplaces (*P* = .001) and restaurants (*P* = .03).

**Table 3:** Effect of Exposure to an Educational Media Campaign on Support for Smoke-Free Policies and Knowledge of the Dangers of Secondhand Smoke (SHS) Exposure, Mobile County, Alabama, 2012^a^

Dependent Variable	No Campaign Exposure		Campaign Exposure		
	%	OR	%	OR (95% CI)	*P* Value
**Support for smoke-free policies**					
Workplaces	79.2	1 [Reference]	86.5	1.9 (1.2–2.8)	.008
Restaurants	77.8	1 [Reference]	88.4	1.9 (1.2–2.9)	.006
Bars	38.5	1 [Reference]	48.2	1.5 (1.1–2.0)	.013
Comprehensive protections for all public indoor areas	68.4	1 [Reference]	74.9	1.2 (0.9–1.8)	.25
**Believe SHS is very harmful**	69.0	1 [Reference]	72.5	1.3 (0.9–1.8)	.22
**Believe SHS causes . . .**					
. . . lung cancer in adults	83.2	1 [Reference]	84.8	1.1 (0.7–1.8)	.53
. . . heart disease in adults	76.9	1 [Reference]	79.8	1.4 (0.9–2.0)	.10
. . . colon cancer in adults	28.5	1 [Reference]	26.6	1.1 (0.7–1.5)	.80
. . . respiratory problems in children	92.6	1 [Reference]	95.7	1.9 (1.0–3.7)	.06
. . . sudden infant death syndrome	39.9	1 [Reference]	38.1	0.9 (0.6–1.2)	.45

Abbreviations: OR, odds ratio; CI, confidence interval.

a Controlling for smoking status, sex, race, and education. The ORs and *P* values were calculated using multivariate logistic regression.

## Discussion

The results of this study offer support for the use of educational media campaigns to broaden support for smoke-free environments and increase knowledge about the dangers of SHS. In assessing the effect of the campaign using logistic regression analyses, we found that those who recalled exposure to the campaign were significantly more likely to support smoke-free workplaces, restaurants, and bars than those who were not exposed. However, in measuring support from precampaign to postcampaign for the full sample, we found an increase in support for only 1 of 4 measures; support for smoke-free bars increased significantly, while support for smoke-free restaurants, smoke-free workplaces, or comprehensive protections in all indoor areas did not change significantly. Support for smoke-free bars is typically lower than support for other smoke-free locations (18–20), allowing more room for improvement. Where support was already high for smoke-free workplaces (79.3%) and restaurants (83.3%), achieving a significant increase in support would be more difficult.

An unexpected finding of the study was that the educational media campaigns had a markedly different effect on black residents than on white residents in changing knowledge about the dangers of SHS. Results comparing knowledge across time show that for black residents, beliefs about the dangers of SHS increased significantly for 4 of 6 knowledge questions, whereas for white residents, there were no significant increases. This finding is surprising, especially because belief in the dangers of SHS was already higher among black residents than white residents on almost all precampaign questions. Greater exposure to the campaign could help to explain this difference; we found that the proportion of black respondents who reported seeing a television advertisement was significantly higher than the proportion of white respondents. Further research may be needed to help explain the discrepancy in these knowledge changes and the potential role of educational media campaigns in targeting demographic groups.

Several limitations to this study should be noted. Our survey used unaided recall of television or radio advertisements as a measure to operationalize exposure to the educational media campaigns. Although this measure has the benefit of being conservative, it is strictly dichotomous and does not account for the effect of varying degrees of exposure to the campaign. Furthermore, we did not attempt to measure the independent effect of the 2 educational media campaigns; thus, findings reflect the effects of both the local and the national campaigns on Mobile County residents. On the basis of their focus group results, MCHD selected less aggressive, colorful advertisements with the principal goal of educating about the dangers of SHS and promoting the message that everyone has the right to breathe smoke-free air in enclosed public places. However, advertisements emphasizing the serious health effects of smoking on individuals are most effective at generating increased knowledge or positive beliefs (21). Therefore, although the Just Breathe campaign aimed primarily to educate about the dangers of SHS in an attempt to change attitudes regarding smoke-free environments, the Tips campaign likely supported MCHD’s second campaign goal of focusing on key health effects of smoking.

Our findings coincide with recent research and evaluation (9,10) suggesting that educational mass media campaigns have the potential to increase support for smoke-free environments. Our study supports the hypothesis that conducting educational media campaigns before the implementation of smoke-free protections can be an effective means of shifting attitudes in favor of these protections. One may infer from this study that among workplaces, restaurants, and bars, attitudes toward smoke-free bars may have the greatest likelihood for change, which is noteworthy, because support for smoke-free bars is typically lower than support for other smoke-free locations. These findings are important because they highlight the potential effects of a public health intervention that should be considered when communities are seeking sustainable, high-impact environmental changes to protect residents from the dangers of SHS. Future research and evaluation should explore the effect of different messages and educational media campaign approaches on knowledge of the dangers of SHS and support for smoke-free environments.
